# Understanding patients' perspective in the use of generic antiepileptic drugs: compelling lessons for physicians to improve physician/patient communication

**DOI:** 10.1186/1471-2377-9-11

**Published:** 2009-03-17

**Authors:** Kore Liow

**Affiliations:** 1 Via Christi Comprehensive Epilepsy Center, University of Kansas School of Medicine at Wichita, 848 N St Francis, 3950 Wichita, KS 67214, USA

## Abstract

**Background:**

Epilepsy is a condition in which consistency of treatment is paramount to successful management and for most patients, effective seizure control can be achieved. Given the severe consequences of even a single breakthrough seizure, patients should be afforded every opportunity to succeed on their given regimens.

**Discussion:**

Some experts argue that global policy on generic antiepileptic drug substitution in epilepsy should be limited – occurring at the discretion of and with careful monitoring by the physician. While the debate continues, physicians still have daily responsibilities to their patients to help them best manage their epilepsy within the context of the current environment – the reality of which may involve switching to a generic antiepileptic drug or navigating various formulations between generics.

**Summary:**

To provide context, this paper first reviews the main "hot button" issues fueling the ongoing generic debate, including a broad overview of the current state of the literature. The main goal however is to provide physicians with a patient perspective on generic antiepileptic drug use in epilepsy as a source of clinically useful, everyday advice to improve communication and increase patient self-advocacy, both of which are necessary for optimal patient outcome.

## Background

Due to increasing healthcare costs, it is increasingly common and beneficial for patients to switch to generic medications. [1,2] The switch for patients with chronic diseases like hypertension and hyperlipidemia could result in cost savings, but epilepsy is different from many other chronic conditions in that it typically responds to treatment in an "all or nothing" manner. Many patients with epilepsy could do well with generic medications, but for other patients, the switch could be devastating. Even a single breakthrough seizure could put the patient at immediate risk for injury, loss of income, driving privileges, or death. [3] Cost-savings gained from lower prescription drug costs may be lost after accounting for emergency room visits or hospitalization, not to mention the personal costs incurred by patients suffering breakthrough seizures. [4,5]

Many newer generation antiepileptic drugs (AEDs) have recently lost patent protection or soon will, serving to further fuel the ongoing debate over the risk/benefit profile of generic substitution in epilepsy treatment. For over 20 years, an ever-increasing collection of anecdotal reports, small retrospective analyses and survey results raising concerns about the interchangeability of generic antiepileptic drugs (AEDs) with their branded counterparts in the treatment of epilepsy has been gathering in the scientific literature. Although overall these reports lack scientific rigor, many neurologists and epileptologists remain concerned that many countries' current bioequivalence standards are inadequate in epilepsy given the reports of breakthrough seizures after a treatment change. [6-10] Furthermore, persons with epilepsy may exhibit a natural wariness toward any treatment switch especially when the mantra of "consistency of treatment" is a familiar goal in trying to achieve and maintain seizure control.

The debate of branded vs generic AEDs will continue until well-controlled studies evaluate the causative relationship between generic substitution and increased risk to patients (either through the occurrence of breakthrough seizures or increased toxicity). This discussion will first provide a brief overview of the factors fueling the ongoing debate including a top-level review of the scientific literature. With the understanding that this debate is likely to continue for some time, the overall objective of this paper is to provide practical "in the clinic" advice to implement in daily interaction with your epilepsy patients. These recommendations are based upon a patient forum in which the goal was to identify ways to improve communication between patient, physician and pharmacist and to develop patient self-advocacy, a realization benefitting both patient and physician.

## Discussion

### Fueling the Debate

#### Bioequivalence Versus Therapeutic Equivalence

In the United States and the European Union, approval of a generic formulation of a drug requires a comparison of the generic form with the corresponding brand drug in small crossover trials in several dozen healthy volunteers (a minimum of 12 are required for European Medicines Agency [EMEA] guidelines). [11-13] Bioequivalence is established through a comparison of the rate of absorption (peak plasma concentration [Cmax] and area under the plasma concentration-time curve [AUC]. The criteria set forth require that the 90% confidence interval of the ratio of the generic to the branded reference compound for the pharmacokinetic parameters mentioned above fall within an acceptance range of 80% to 125%. If a generic drug is deemed to be bioequivalent and has the same active ingredient as the branded drug with the same dose availability and routes of administration, the drug is approved as therapeutically equivalent and substitution is allowed without risk of toxicity or diminished efficacy.

This standard for evaluating bioequivalence has a number of critics who are quick to point out that these bioequivalence studies are typically conducted with single doses on a relatively small number of healthy, young, male volunteers. [14] In "real life," generic preparations will be given to elderly and pediatric patients (a substantial portion of the epilepsy patient population) as well as those taking a number of other medications and potentially with concomitant conditions. In addition, the boundaries of "acceptable" variability may be tested when considering that often numerous versions of a generic product are on the market, raising the possibility of receiving one drug at the top of the "acceptable" range and then being switched without notification to a second generic that was approved at the bottom of the "acceptable" range. [14] In direct response to these concerns, the Danish Medicines Agency narrowed their definition of acceptable bioequivalence for a select group of generics, including AEDs, to fall within a 90% to 111% range of the reference compound. [15,16] Further complicating the situation, regardless of the exact definition of bioequivalence, is the reality that individual patient differences can lead to therapeutic nonequivalence. Indeed, a recently published study of nine outpatients in Denmark who had reported problems following a change in the preparation of lamotrigine found that, even with the narrower bioequivalence requirements for mandatory substitution in Denmark, some patients experienced serious clinical consequences (eg, relapse of seizures) in association with a change in preparation that corresponded to significant alterations in plasma levels. [17] Though these results may be biased due to the retrospective nature of the study, they do underscore the problems that can occur with AED formulation shifts due to individual patient differences. Finally, though beyond the scope of this paper, another broad category perpetuating the debate is the ongoing discussion of the role that individual drug characteristics may play in establishing true bioequivalence (eg, those drugs with a narrow therapeutic range, nonlinear kinetics, or those in extended release formulations). [18]

#### Challenging the status quo

Regulating agencies contend that bioequivalence establishes therapeutic equivalence; however this stance is in contrast to several published accounts of breakthrough seizures and increased toxicity upon switching to or between generic formulations. [3,7,19-22] The disparity between government policy and what physicians and patients may be experiencing "in the real clinical scenario" has led to many calls to action within the literature. [12,19,23-26] as well as to the development of position statements by scientific and medical organizations. [27-29] The most recently published position statement on the coverage of anticonvulsant drugs for the treatment of epilepsy issued by the American Academy of Neurology (AAN) states unequivocally that the AAN "opposes generic substitution of anticonvulsant drugs for the treatment of epilepsy without the attending physicians approval." Furthermore, the recommendations summarize that the use of newer generation AEDs should not be limited (eg, through "fail first" policies or high co-pays). The AAN position statement also lays out the rationale for its opposition of prior authorization requirements by private and public formularies, namely that it is one method formularies may use to limit access to AEDs. The position taken by the AAN is consistent with guidelines prepared by other governing agencies as well, though, as will be explored later, there are regional differences.

The purpose of these and other recommendations is not to disparage all generic use. In fact, generic use in *de novo *patients or when monitored carefully following a treatment switch can be an important cost-saving measure and may often occur with success. Rather, at the heart of these recommendations is the goal of continuity of care for epilepsy patients, a core principle in managing this disease. Epilepsy is a condition that is controllable, and patients should be given every chance to succeed with their treatment regimens. Patients not receiving optimal care are at risk for breakthrough seizures, the consequences of which can be severe as well as costly (financially and emotionally), including driving accidents, loss of work, increased emergency room visits and diminished quality of life. [5,14,28,30]

#### Strength of the Literature

Published reports of breakthrough seizures accompanying a switch to a generic AED formulation begin to appear in the literature as far back as the 1980s. [21,31] Early published reports of double-blind cross-over studies such as those by Hartley et al (N = 23 children), Oles et al (N = 40) and Mikati et al (N = 10) attempted to more rigorously evaluate the potential risk of a treatment switch in epilepsy. [20,32,33] Even though these studies did not identify decreased efficacy following a treatment switch, the ability to generalize these and other crossover study results to widespread generic policy is difficult given that different drugs were evaluated, overall patient numbers were low, and no consistent effect on serum levels was observed.

In spite of these studies and a continued presence of this issue in the scientific literature over an extended period of time, evidence-based conclusions on the appropriateness of generic AED substitution have been hampered by a lack of rigorous, well-controlled studies. To evaluate what has been most recently published, a Medline search was conducted using search terms "epilepsy" and "generic," and limited to English language publications from the last 5 years. After eliminating irrelevant articles, the search revealed 4 claims studies, 4 economical analyses, 16 reviews/opinion pieces, 4 serum level studies, and 6 surveys. Similar to the profile of the publications over the past two decades, most of these publications supported the limitation of generic substitution in epilepsy treatment, yet for the most part these were uncontrolled observations. Two of the more compelling studies most recently published (Le Lorier 2008, N = 671 and Andermann 2007, N = 1354) evaluated medical/pharmacy claims data to compare rates of switchback among users of AEDs compared to other therapeutic areas. In both studies, a higher propensity to switch back to branded medications was observed for AEDs compared to other therapeutic classes. [24,34] In addition, both of these studies showed a statistically significant increase in AED dose for those patients not switching back.

As a whole, the data within the scientific literature are insufficient to support policy changes in that rigorous, well-controlled studies are lacking; however, they do serve to convey the long-held concerns shared by physicians and patients regarding the connection between the use of generic AEDs and the occurrence of breakthrough seizures. These data also underscore the need for further study and a definitive examination of cost benefit in epilepsy. One of the biggest advantages to generic drug use is the short-term cost-savings it affords; however, while the switch to generic AEDs may be successful and cost effective in most patients, effective seizure control cannot be sacrificed based solely upon financial considerations. It remains unclear if short-term cost savings are sustained after accounting for the occurrence of breakthrough seizures and/or increased toxicity that may accompany generic substitution in individual patients. [4]

Physicians have daily responsibilities to their patients to help them best manage their epilepsy within the context of the current environment – the reality of which may involve switching to a generic drug or navigating various formulations between generics. In order to truly effect change, we must start with our own patients by improving communication and providing them with the necessary tools in order to self-advocate. The following section provides useful insight and suggested tips for the physician to pass along to his or her patient based upon feedback from those who should know best – our patients.

### The Patient Perspective

In a physician-moderated patient forum held March 30, 2008, in Washington, D.C., 20 epilepsy patients and caregivers shared perspectives, anecdotes, and advice on the challenges they have faced and continue to navigate in their attempt to best manage their seizure control with AEDs. It should be noted that participants were active members in the U.S.-based and industry-sponsored Epilepsy Advocate program and therefore do not necessarily represent an equal sampling of the epilepsy patient population; however, the content of their discussion revealed common themes and challenges faced by persons with epilepsy everywhere as well as the need for improved communication with our patients.

My objective therefore was to provide patient insights to physicians to help improve the level of care. The most salient features of the sections below are organized by levels of patient interaction. Due to the fact that patients participating in the forum are in the US, some of the specific recommendations may only apply to a US patient population and are so noted. Because the main goal of this paper is to provide physicians with the tools necessary to help improve the lives of their epilepsy patients, the discussion points are further noted in a patient/physician communication tip sheet (Table 1).

**Table 1:** Patient/Physician Communication Tip Sheet: Improving Patient Experience with Generic AEDs

**Discuss with your physician **whether you are a suitable candidate for AED substitution or may be at risk for seizure breakthrough.

**Learn the typical signs of over and under treatment **with your AED or a substituted agent:
• Over treatment
- Dizziness, sedation, rash
• Under treatment
- Breakthrough seizures, auras

**Honesty is the best policy! **Accurately report dosing history, including missed doses, to your physician and any associated side effects or breakthrough seizures. To assist with this effort, keep a diary to record seizures and side effects.

**If possible, frequent the same pharmacy **to fill your prescriptions and become familiar or friendly with the neighborhood pharmacist.

**"Know your pills" **whether branded or generic versions – the size, the color – and inspect your medication and its label before leaving the pharmacy. Ask questions, if necessary!

**Per the "Patient's Bill of Rights," **know you have a right to receive the medication you expect to receive; if you have been given a medication (i.e., generic substitution) that is not what you expected and you feel uncomfortable accepting, it is within your right to purchase the exact pills you have been taking. Buy just one pill if necessary to make it through to your next dose and give yourself time to consult your physician.

**Learn the details of your insurance policy**. If it has been determined by your physician that you remain on a branded drug not covered by your policy, it is your right to request the drug be approved and added, and if necessary, appeal a decision if request is initially rejected.

**Learn your state's laws regarding generic substitution **and "DAW" prescriptions, and if necessary, call your legislator or write a letter to your congressperson in cases where you feel state laws must either be enacted or changed. (Visit for more information about state laws governing AEDs.)

**Inform your doctor of side effects **and breakthrough seizures following AED substitution and ask that they be reported to MedWatch, the FDA's safety information and adverse event reporting program. (For information, call 1-800-FDA-1088 or visit the web site at .)

### Communicating Practical Advice to Your Patient

#### Level 1: Patient/Physician Relationship

Optimum epilepsy treatment begins at the most basic of the relationships: the patient and their physician. Indeed, only through the establishment of good communication between these two parties can self-advocacy be attained.

To that end, there are many important facts that patients need to be informed of that affect their ability to best assist in their own individual treatment management.

#### Challenges

Given the multitude of newer and older antiepileptics used as primary and adjunctive therapy – both in brand and generic formulations – it becomes even more important and perhaps confusing for the epileptic patient to understand the reasoning behind their physician's selection of treatment. It is paramount that a physician discusses his/her rationale with the patient in terms of AED selection and the possible side effects and symptoms of over/under treatment, thus empowering their patient to become a partner in the management of their epilepsy with the goal of a positive treatment outcome.

Studies suggest that patients who engage in open dialogue with their physician tend adhere to their AED regimen. [35-38] Therefore it is advised that physicians stress the importance of patient compliance and drug adherence to patients and caregivers as it is paramount to treatment success whether they are taking brand, generics, old or new AEDs.

#### Tell your patient

It is important your patient understand the treatment selected and the expectations you have for this treatment in terms of attempting to effectively manage their epilepsy seizure-free, particularly in the context of possible introduction of generic substitution.

Given that many newer generation AEDs have recently come off patent, or soon will, and the increasing possibility in the patient's future of generic substitution either by way of mandatory requirement or voluntary selection, the patient must be able to recognize the typical warning signs of over/under treatment of AEDs.

While many epileptic patients are knowledgeable about the typical side effects of the drugs themselves, they may not be able to recognize at first glance the effects of over/under treatment. For example, dizziness and sedation are two possible signs of over treatment, while having breakthrough seizures or auras could be signs of under treatment. [39,40]

It is within the context of the issue of physician and patient communication that compliance and adherence must be discussed. Indeed, as like recognizing over/under treatment, compliance – or lack thereof – strongly affects a patient's success in terms of optimum treatment and can undermine the critical physician/patient relationship.

To effectively discuss compliance and adherence some physicians may need to reevaluate their patient communication. In a recent AED adherence survey of epilepsy patients and physicians, 31% of physicians believed that they "spend a lot of time talking with their patients about epilepsy," whereas only 14% of patients agreed with that statement. Additionally, patients who reported a trusting relationship with their physician were more likely to be adherent and willing to discuss missed doses. [41]

From the patient's standpoint, an informal industry-sponsored internet-based survey of 451 epilepsy patients found that patients oftentimes fail to report non-compliance to their doctors. Although 71% of patients agreed that missing a dose of their AED – be it branded or generic – is a serious issue and can lead to seizure, only 41% of patients reported that they immediately take the dose upon remembering. (To note, 37% of patients who reportedly missed a dose had at least one breakthrough seizure, of which 38% required a visit to the emergency room.) Further, 43% did not inform their physician immediately that they had missed a dose, and 41% waited until their next appointment to do so. [42]

It is important for physicians to tell their patients that "honesty is the best policy." It is imperative that a patient or caregiver accurately report to their physician dosing history, including missed doses, in an appropriate timeframe. For the physician's part, you must remember that in an effort to empower your patient to be your ally in their treatment and optimum health, they should keep a seizure diary and learn to trust that their communication of missed doses or lack of drug adherence will serve to strengthen your partnership and that the result will be a better, more honest base on which to build and improve the patient's quality of life in their effort to remain seizure-free be it through use of generic or branded AEDs.

### Keeping Your Patient Safe

#### Level 2: Patient/Pharmacy Interaction

Given the sheer number of prescription drugs available – both in brand and generic form – which is growing every day, it is has become increasingly important for patients to be aware of policy issues and everyday examples of safe practices relating to their interactions at the pharmacy level.

#### Challenges

Further complicating the issue is that any given generic may have many different manufacturers. (For example, 17 different generic versions of zonisamide are available.)[12] Each of these generics will have unique formulation differences; for pharmacists, this means a continuously changing pool of medications – both branded and generic – and manufacturers depending on current contractual agreements.

Finally, treatment decisions can potentially be impacted by pharmacy as use of generics and preparation shifts in the treatment of newly diagnosed patients who continually experience breakthrough seizures could lead physicians to erroneous assumption that a patient's seizures are resistant to drug. [43]

#### Tell your patient

As a physician, it is important to tell your patient that when he/she walks through the door of their local pharmacy or calls in their AED prescription refill request, there are several ways in which they may help manage their treatment and ensure their needs are best being met, particularly given the increased potential for generic substitution at some point in a patient's life.

First, it is imperative for the patient to attempt to establish a trusting and honest relationship with their pharmacist. Advise your patient to, if possible, frequent the same pharmacy to fill their prescriptions and become familiar or friendly with their neighborhood pharmacist. "I think it's crucial that you know your pharmacist and they get to know you and let them know your story, especially when it's as critical as epilepsy."[44]

Second, physicians must advise their patients to "know your pills" (or liquid medication). To best ensure safety, patients must make a point of knowing what each of their medications looks like whether branded or generic versions – the size, the color – and always double-check the label to confirm accurate patient name and address, prescribing physician, drug dispensed, manufacturer, dosing instructions, etc. "I open the bag right at the pharmacy and take a close look at them and if those aren't the pills I've been taking, I flat-out refuse them."[44]

U.S. physicians also should make their patients aware of the "Patient's Bill of Rights," which states in effect that a patient has the right to make sure that they are getting the medication that they expect to get. [45]

And if for some reason (i.e., generic substitution) your patient has been given a medication that is not what they expected or typically receive – and feels uncomfortable accepting, the patient should know that it is medically advisable and perfectly within their right to purchase the pills they have been taking, even if in a low quantity. This will allow the patient to meet their dosing schedule for a few days in order to provide enough time to be able to follow up with their physician. Indeed, patients must know it is acceptable to buy just one pill if necessary to make it through to their next dose and give them time to consult their physician.

Lastly, the patient may wish to check with their healthcare provider in order to inquire as to how many days must transpire between refill orders; knowing this, for instance, will help the physician, patient, and pharmacy manage and sustain an adequate supply of their AEDs by requesting refills with enough time available to sustain a supply delay in the instance their medication is out of stock and the pharmacy needs to order. [44]

### Appreciating a Patient's Residence

#### Level 3: Regional Differences

Health care systems vary greatly from country-to-country, as well as state-to-state in the U.S., but the one thing they have in common is that they are under increased pressure to control the costs of prescription drugs and other services, which has prompted efforts to strongly "encourage or mandate the substitution of medications with generic preparations."[13]

Further complicating the issue is the fact that private insurance companies and public health services have varying and unequal coverage. In the U.S., laws governing generic substitution vary by state and apply regardless of whether a patient's payment is through a public insurer (i.e., Medicaid) or a private insurance company. [46] As each of the states carries its own set of rules and regulations thus adding to the confusion, it is, therefore, imperative for patients to understand and appreciate the role that residence plays in their "continuity of care" (Table 2).

**Table 2:** Drug-Substitution Laws Applicable to U.S. Patients

**Type of Law**	**States and Territories**
**Mandatory/permissive substitution:** States generally either permit or mandate that the pharmacist substitute a generic version of a prescribed drug if all prescription requirements are met.	**Mandatory: **FL, KY, **MA**, MN, MS, **NJ**, NY, PA, PR, **RI**, **WA**, WV **Permissive: **AL, AK, AZ, AR, CA, CO, CT, DE, DC, GA, GU, HI, ID, IL, IN, IA, KS, LA, ME, MD, MI, MO, MT, NE, NV, NH, NM, NC, ND, OH, OR, SC, SD, TN, TX, UT, VT, VA, WI, WY
	
**State Drug Formulary: **Some states provide a positive (drugs are equivalent and interchangeable) or a negative (drugs are not equivalent and not interchangeable) formulary to guide appropriate substitution.	**Positive: **DE, DC, FL, HI, IL, MA, NE, NV, NH, NJ, NY, TN, UT, VA, WI, **Negative: **AR, KY, MN, MO, NC
	
**Patient consent/notification requirement: **Most states require patient consent for, or notification of, substitution.	**Required: **AK, AZ, CA, CO, CT, DE, DC, FL, GA, HI, ID, IL, IN, IA, KS, KY, ME, MD, MI, MN, MS, MO, MT, NE, NV, NH, NY, ND, OH, PA, PR, SC, SD, TX, UT, VT, VA, WV, WI, WY **Not Required: **AL, AR, GU, LA, **MA**, **NJ**, NM, NC, OR, **RI**, TN, **WA**
	
**Cost savings requirement: **Most sates require that the drug dispensed be less or no more expensive than the drug prescribed and that some of the cost savings be passed on to the purchaser.	**Less or no more expensive: **AK, AR, CA, DC, GA, GU, HI, ID, IL, KS, KY, ME, MD, MA, MS, MO, NV, NH, NJ, NY, NC, ND, OH, OR, PA, PR, RI, TN, TX, VT, VA, WI, WY **Savings passed on: **CO, CT, DE, FL, IN, IA, ME, MD, MI, MN, MT, NE, NM, RI, TN, WA, WV **Requirement not mentioned: **AL, AZ, LA, ME, PR SC, SD, UT
	
NTI drugs recognized as special category	**Recognizes NTI: **KY, NC, PA, SC, TN

**Boldface states and territories **= mandatory generics substitution without patient consent/notification

Underlined states and territories = permissive generics substitution without patient consent/notification

NTI: narrow therapeutic index

Adapted from [47-49]

#### Challenges

In regions where generic substitution in mandated, physicians may have to "jump through extra hoops" to ensure their patients receive the brand drug, if so prescribed. In the U.S., all states have laws requiring that brand drugs be dispensed if so ordered by a prescribing physician, but most states allow pharmacists to make a generic substitution if a prescription is not specifically marked "dispense as written" (DAW). Further, in more than a dozen states, generic substitution is mandated unless a physician specifically marks a script "DAW," and in four states generic substitution is mandated without requiring patient notification. [47-49]

Differing regional laws that govern pharmacy mandates and requirements makes it difficult for all parties involved, and further underscores the importance of good communication between physicians and their patients.

#### Tell your patient

First, you must relay to your patient how important it is to know their insurance plan, whether private or public. Having knowledge of their healthcare policy will allow the patient to be best able to communicate their coverage accurately and effectively as to how it may relate to their AED treatment.

In terms of the potential for generic substitution, if a physician has determined that it is imperative for their patient to remain on a branded drug to help increase likelihood of seizure-free status, the patient must know that it is not only well within their right, but also their obligation to advocate for the AED of their choice.

In some instances, it may require a patient to work with their physician to draft a letter or letters to their private insurance company or national healthcare provider requesting that a particular drug be approved and added to their policy's coverage. In these cases, it is beneficial for your patient to know their provider's process and timeline as each insurer may have slight differences in process.

In addition, it is important for a patient to understand that such a request can sometimes be accompanied by a battle that ultimately is well worth waging. A patient must be prepared to appeal an initial decision and demand that their case be heard before a Review Board should the insurance provider deny the first request.

For patients with private insurance, the entire appeals process will likely take more than submitting a single letter, and oftentimes many patients give up on the process prematurely. For example, in the U.S., 67 percent of appeals that are denied the first time never get presented another time. Physicians must encourage their patients to exhaust all levels of appeal. Even if an appeal is ultimately denied, the insurance company could reverse its decision years later and retroactively cover the costs. [50]

Regardless of your country of residence, the lack of definitive studies on generic versus branded AEDs has created an environment where physicians, patients, and pharmacists are functioning without a global standardized set of guidelines. Unfortunately, this can also lead to even more confusing regional differences. For example, 40 U.S. states, legislatures are considering laws that would require pharmacists to contact a physician to substitute a generic epilepsy drug, regardless of whether it was marked DAW or not. Further, legislators in 18 states are considering laws that would ban generic substitution for epilepsy drugs. [46]

In all cases, physicians must make clear to their patients that decisions related to their drug treatment should be made on a case-by-case basis with their personal well-being first and foremost in mind. Mandatory generic substitution is just as objectionable for pharmacy as is legislation to restrict generic substitution. [50]

### A View from the Top

#### Level 4: The National/International Climate

In order for patients to gain a complete understanding of the "big picture," physicians must communicate to their patients the impact of national policy and economic considerations and how these may affect the patient's own individual care.

#### Challenges

Despite the fact that the issue of generic substitution of AEDs is being increasingly highlighted in the scientific literature and medical field, the possible ramifications, national and international reporting efforts, and pharmacoeconomic analyses may not be widely known at the community level.

#### Tell your patient

In an effort to empower patients towards self-advocacy, physicians are advised to make their patients aware of the following considerations.

First, it is absolutely necessary for patients to report any breakthrough seizures or side effects following AED substitution to their physicians. U.S. physicians can and should report these side effects and any adverse events to MedWatch, the FDA's safety information and adverse event reporting program, a process which takes 20 to 40 minutes. (For information, call 1-800-FDA-1088 or visit the web site at .)

Alarmingly, despite the fact that 65% of physicians have cared for patients with breakthrough seizures as a result of switching from branded to a generic agent, this finding is not reflected in the scientific literature or MedWatch. [12]

Physicians who care for patients who have breakthrough seizures or side effects related to generic substitution should make every effort to obtain complete data, including: AED blood level at the time of the breakthrough seizure or side effect; a corresponding AED blood level (preferably at the same time of day, in the same clinical laboratory) once the patient is again stable on the brand or initial generic AED; detailed confirmation of compliance; rechallenge with the substituted generic AED at a future time (if the patient is willing). [12] After at least five half-lives a blood level at the corresponding time should be obtained. Then the patient should return to the baseline preparation to minimize the chance of a second breakthrough seizure or side effect. [12]

Moreover, a patient must know that they may feel a slight difference day-to-day with varying medications, not only when switched from branded drug to generic, but also from one generic to another, and it is imperative they notify their physician to report such side effects.

Lastly, more accurate reporting in the clinical setting may impact and lead to a better understanding of the pharmacoeconomic literature. To date, recent economic models have suggested that short-term savings realized by generic substitution may be erased by rising costs elsewhere. [2] Therefore, it is necessary for all parties involved to appreciate the associated real or potential costs associated with a treatment switch (i.e., monitoring plasma levels and the cost of managing loss of seizure control). [2,10,51] The economic cost of poorly controlled epilepsy is enormous, and the most cost-effective intervention is an AED that provides total seizure control. [5]

Of course, balance will vary between different AEDs and different countries and may not always favor use of generics. For example, a pharmacoeconomic modeling study performed in Spain demonstrated that if 9% of patients with epilepsy taking brand-name carbamazepine were switched to a generic, this would not be cost-effective. The annual individual patient cost would rise by €38 as compared with treating these patients with brand-name carbamazepine, and overall spending on health care in the country would increase by €2,748,000, principally due to increased spending on consultations and hospitalizations for loss of seizure control. [51]

In another example, a questionnaire-based survey of neurologists in the United States also provided clues to the cost effectiveness of generic AED switching. Of a total sample of 301 neurologists, 81.4% observed an increase in the frequency of seizures (67.8%) or adverse events (56.0%) after patients switched from a branded drug to a generic version. This necessitated more consultations, emergency room treatment, and hospitalizations, resulting in an additional incremental cost of US $675,004, mainly from the cost of hospitalizations. This incremental cost offset to a large extent the savings derived from use of a less expensive generic. [10]

To reiterate, what may be considered by some as a savings now (i.e., generic), may lead to paying later (i.e., hospitalization for treatment associated with breakthrough seizure, toxic side effects).

"If they could just think of it this way: Switching from a brand to a generic may save $50 per month, but one hospitalization due to a seizure breakthrough may cost $4,000–$6,000....and that'd take decades to recoup."[44]

## Summary

Given the devastating consequences of even a single breakthrough seizure on the quality of life of epilepsy patients, it is crucial that physicians treating epilepsy patients begin to educate and engage patients in discussion in regards to the use of generic antiepileptic drugs. It is equally important for physicians and patients to ensure that there is "continuity of care" of the same medications that have been controlling their seizures.

Furthermore, should a switch in their antiepileptic medication be made, whether from brand to generic or one form of generic to another, it must be done so in a safe manner, i.e. with the full consent and knowledge of both the physician and patient to ensure that any sign of over or under treatment can be promptly identified and corrective action taken. Finally, physicians and patients alike must be aware that only through improved communication and increased patient self-advocacy can optimal patient outcome be realized.

## Abbreviations

AEDs: antiepileptic drugs; EMEA: European Medicines Agency; Cmax: peak plasma concentration; AUC: area under the plasma concentration-time curve; AAN: American Academy of Neurology; DAW: dispense as written.

## Competing interests

Dr. Liow has received research grant/support from National Institutes of Health, Centers for Disease Control, NeuroPace, Inc., Johnson & Johnson, Schwarz Biosciences, Inc., Eisai, Inc., UCB, Inc., GlaxoSmithKline, and Ortho-McNeil Pharmaceutical, Inc. He also has received consulting and speaker's bureau fees and honoraria from Pfizer Inc and UCB, Inc.

## Authors' contributions

KL Moderated the patient forum, drafted the manuscript, read and approved the final manuscript

## Pre-publication history

The pre-publication history for this paper can be accessed here:


